# Mirizzi Syndrome: The Uncommon and Overlooked Surgical Cause of Obstructive Jaundice

**DOI:** 10.7759/cureus.85611

**Published:** 2025-06-09

**Authors:** Karan Yagnik, Sandeep V Kotnani, FNU Payal, Jilliane Unas, Rutuja Challawar, Anoohya Vangala, Doantrang Du, Dharmesh Kaswala

**Affiliations:** 1 Internal Medicine, Monmouth Medical Center, Long Branch, USA; 2 Internal Medicine, RWJBarnabas Health, Long Branch, USA; 3 Gastroenterology, Monmouth Medical Center, Long Branch, USA

**Keywords:** mirizzi syndrome, obstructive jaundice, open laparotomy vs laparoscopy, surgical obstructive jaundice, unexplained abdominal pain

## Abstract

We present a case of Mirizzi syndrome in a patient who exhibited abdominal pain along with signs of obstructive jaundice. The minimally invasive approach was complicated, necessitating an open laparotomy, which ultimately prolonged the hospital stay. A female in her early 60s with a history of rheumatoid arthritis (on methotrexate) and hypothyroidism presented to the emergency department with jaundice as her chief complaint. Upon arrival, the patient was vitally stable. The only pertinent finding was scleral icterus. Laboratory results revealed AST of 263 U/L, ALT of 233 U/L, ALP of 1246 U/L, GGT of 342 U/L, total bilirubin of 5.8 mg/dL, and direct bilirubin of 4.6 mg/dL. Abdominal ultrasound showed dilated intrahepatic and extrahepatic bile ducts, with a common bile duct measuring 16 mm, raising concern for biliary obstruction. Magnetic resonance cholangiopancreatography (MRCP) showed marked intrahepatic ductal dilation resulting from extrinsic compression of the common hepatic duct (CHD) by impacted gallstones at the gallbladder neck, findings that are consistent with Mirizzi syndrome. Hence, endoscopic retrograde cholangiopancreatography (ERCP) was deferred and she underwent subsequent laparoscopic cholecystectomy, which turned into open surgery, and ended up getting bile duct resection with hepaticojejunostomy. Her postoperative course got complicated with partial small bowel obstruction, which was managed conservatively. Mirizzi syndrome presents a formidable diagnostic and therapeutic challenge. Our experience with this particular case reinforces that laparotomy offers a safer and more effective approach for managing Mirizzi syndrome in similar circumstances.

## Introduction

Mirizzi syndrome is a rare biliary disorder caused by the compression of the common bile duct (CBD) due to a gallstone lodged in the cystic duct or Hartmann’s pouch. First described by Pablo Luis Mirizzi in 1948, it typically presents with cholestatic jaundice and abdominal pain, often resembling choledocholithiasis. Women comprise 50% to 77% of cases, likely due to a higher incidence of gallstones in older women, although some literature suggests no gender predisposition [1].

Mirizzi syndrome presents diagnostic and therapeutic challenges, often due to anatomical variations and complications. While ultrasound is typically the initial imaging modality, definitive diagnosis usually requires advanced imaging such as endoscopic retrograde cholangiopancreatography (ERCP) or magnetic resonance cholangiopancreatography (MRCP) [2]. Open cholecystectomy is preferred because laparoscopic surgery poses a higher risk of bile duct injury and often requires conversion to an open procedure [1]. Inadequate management of Mirizzi syndrome may lead to severe complications, such as cholecystocholedochal or biliary-enteric fistulas and gallstone ileus. This article presents a case of Mirizzi syndrome in a patient with abdominal pain and obstructive jaundice, whose hospital stay was prolonged due to surgical complications.

## Case presentation

A 62-year-old female, weighing approximately 50 kg, with a history of rheumatoid arthritis (on methotrexate 5 mg three times per week) and hypothyroidism, presented to the emergency department with the primary complaint of jaundice. She had experienced abdominal discomfort, tiredness, and nausea a month prior, initially attributed to food poisoning. Jaundice developed gradually after these symptoms subsided. Outpatient liver function tests revealed significant abnormalities, prompting her referral for further evaluation.

Upon arrival, her vital signs were stable. Physical examination was notable only for scleral icterus; otherwise, her abdominal examination was benign including negative Murphy's sign. Laboratory workup showed AST of 263 U/L, ALT of 233 U/L, ALP of 1246 U/L, GGT of 342 U/L, total bilirubin of 5.8 mg/dL, and direct bilirubin of 4.6 mg/dL. WBC count was 11.6 × 10⁹/L, lipase was 48 U/L, and the hepatitis panel was negative. Tumor markers were unremarkable (CA 19-9 <2 U/mL, AFP 5.9 ng/mL, CEA 0.94 ng/mL).

**Figure 1:**
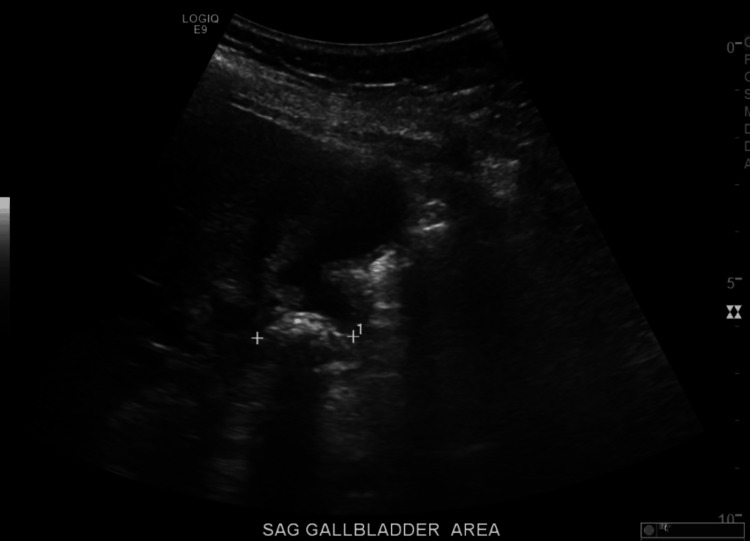
Ultrasound abdomen showing gallbladder stone with dilatation of the common bile duct.

**Figure 2:**
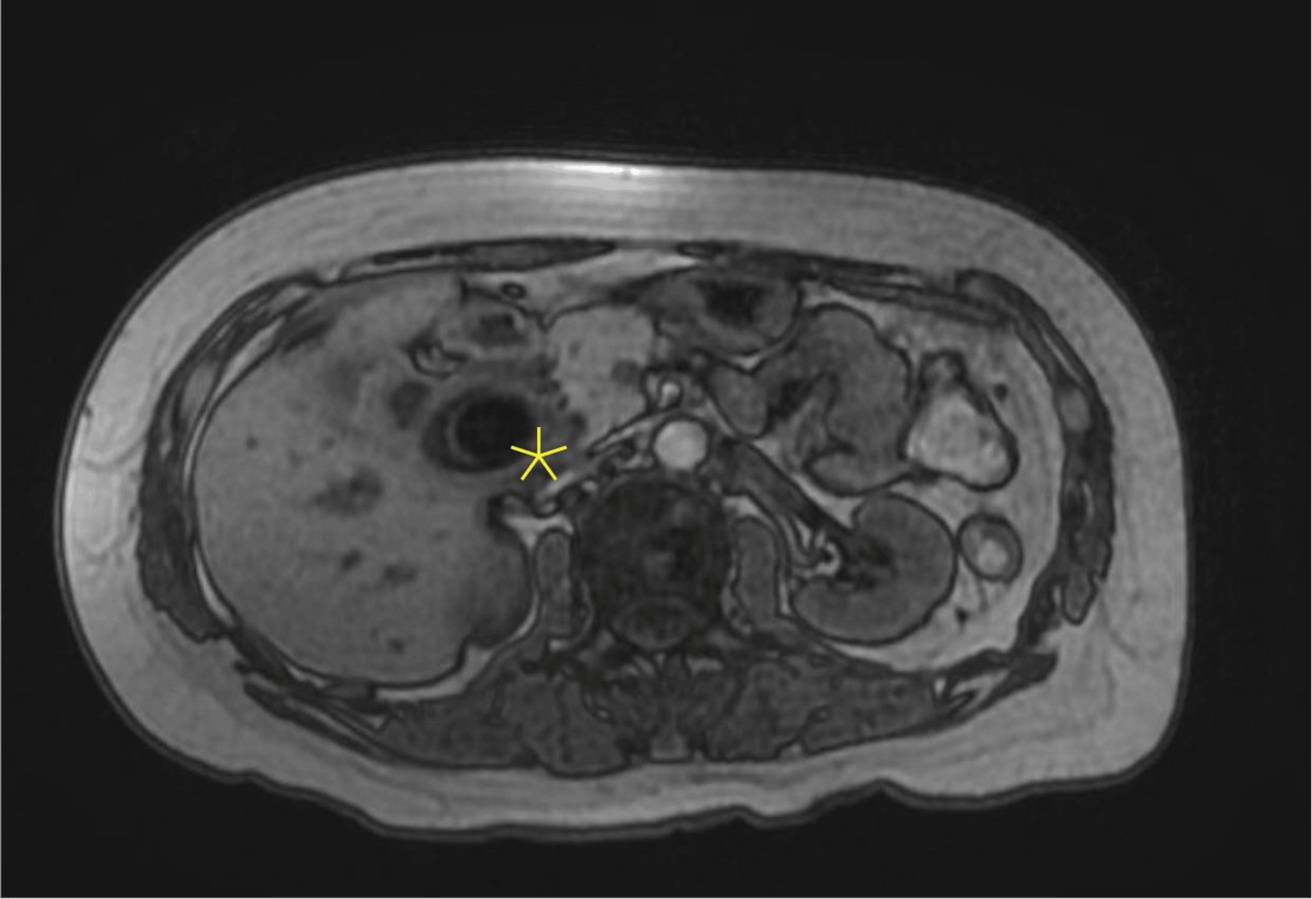
Magnetic resonance cholangiopancreatography (MRCP) showing dilated common hepatic duct due to impacted gallstone at the gall bladder neck.

The abdominal ultrasound demonstrated dilation of the intrahepatic and extrahepatic bile ducts and CBD measuring 16 mm (Figure 1), without any evidence of acute cholecystitis. Subsequent evaluation with MRCP revealed significant intrahepatic ductal dilation due to extrinsic compression of the common hepatic duct (CHD) by impacted gallstones at the gallbladder neck (Figure 2), confirming a diagnosis of Mirizzi syndrome. Hence, emergent ERCP was deferred due to extrinsic compression of the CHD to avoid iatrogenic injury. Later, she underwent a laparoscopic cholecystectomy, which was converted to open surgery due to an inflamed gallbladder adherent to the duodenum. While taking down the adhesions, a small fistula was found extending into the duodenal wall. The procedure ultimately involved bile duct resection and hepaticojejunostomy. Postoperatively, liver function tests improved. A fluoroscopic upper GI single-contrast study on postoperative day 3 showed no evidence of a leak. However, the patient’s hemoglobin levels dropped despite receiving two units of packed red blood cells. Consequently, a CT angiography (CTA) of the abdomen and pelvis was performed on postoperative day 5, which revealed no active bleeding. A subsequent fluoroscopic small bowel study indicated a partial obstruction at the anastomosis, which was managed conservatively with nasogastric tube placement and bowel rest. She was eventually discharged home safely without further complications. Frozen section biopsies were negative for gallbladder cancer.

## Discussion

We present a case of Mirizzi syndrome diagnosed through imaging in a patient with obstructive jaundice. Although postoperative recovery was prolonged, the patient was ultimately discharged in a stable condition. Preoperative diagnosis is often challenging. Mirizzi syndrome is found in approximately 0.1% of patients with cholelithiasis and 0.7% to 25% of patients undergoing cholecystectomy, with no significant gender predisposition, though incidence increases with age, and no specific ethnic prevalence is noted [3].

Mirizzi syndrome typically presents with painless obstructive jaundice, as seen in our case, and can mimic acute or chronic cholecystitis. However, our patient was immunosuppressed due to long-term methotrexate use; therefore, a normal WBC count was not a reliable indicator. Ultrasound may reveal gallstones in the gallbladder infundibulum compressing the CBD, resulting in intrahepatic bile duct dilation. In contrast, chronic cholecystitis is characterized by dull right upper quadrant pain, often triggered by fatty foods, along with nausea, vomiting, and bloating. Patients commonly exhibit right upper quadrant tenderness and a positive Murphy’s sign, typically experiencing discomfort without acute illness; advanced cases may present with more pronounced symptoms and elevated bilirubin levels. Diagnosis generally begins with an abdominal ultrasound or CT scan, while MRCP is used for confirmation, as demonstrated in this case. If an obstruction in the CBD is identified on MRCP, ERCP is considered the gold standard for further evaluation due to its therapeutic potential. However, Mirizzi syndrome is frequently misdiagnosed as a simple CBD stone or overlooked during preoperative assessment.

Surgery is the primary treatment for Mirizzi syndrome, addressing the inflamed gallbladder and impacted stone. During surgery, if Mirizzi syndrome is identified, a cholangiogram should be performed to confirm the diagnosis and evaluate the biliary anatomy before further action [4]. For patients who are not suitable for surgery or with concurrent cholangitis, ERCP may be used as the primary treatment [5]. Additionally, surgeons should maintain a high suspicion of gallbladder cancer and perform a frozen section, as gallbladder cancer is found in about 5% to 28% of patients with Mirizzi syndrome [6].

Types of Mirizzi syndrome and approaches: Type I, no fistula present; Type IA, presence of the cystic duct; Type IB, obliteration of the cystic duct; Types II to IV, cholecystobiliary fistula present; Type II, defect smaller than 33% of the CHD diameter; Type III, defect 33% to 66% of the CHD diameter; Type IV, defect larger than 66% of the CHD diameter; Type V, cholecystoenteric fistula.

Subtotal cholecystectomy is the preferred treatment for Mirizzi syndrome types I-III. In Type I, laparoscopic dissection may be attempted, converting to open surgery if necessary. While laparoscopic techniques are possible for Types II-V, open surgery is often required due to dense adhesions and inflammation as seen in our case with type V Mirizzi syndrome [7,8]. Laparoscopic treatment remains controversial; a systematic review found it successful in 59% (73/124) of cases [9]. A retrospective series showed laparoscopy attempted in 15 of 35 patients, with a 67% conversion rate to open surgery [10]. Based on our case, we consider laparotomy superior. If no fistula is present, the gallbladder stump is closed with absorbable sutures, but biliary-enteric anastomosis may be needed for Types III-V.

## Conclusions

Mirizzi syndrome poses a significant diagnostic and therapeutic challenge due to its diverse clinical presentations and complex anatomy. Our experience with this case highlights that open surgical intervention, particularly laparotomy, may be a safer and more effective option in complex presentations of Mirizzi syndrome. We also emphasize sending frozen sections to rule out gallbladder cancer in patients with Mirizzi syndrome.
